# Supplementary material for: Comparison of the Utility of RP-TLC Technique and Different Computational Methods to Assess the Lipophilicity of Selected Antiparasitic, Antihypertensive, and Anti-inflammatory Drugs
Source: Molecules. 2019 Sep 2;24(17):3187. doi: 10.3390/molecules24173187 (PMC6749294; doi:10.3390/molecules24173187)

S values in accordance with Soczewiński-Wachtmeister's equation

S value from Soczewiński's Equation*	Antiparasitic drugs:			
	Metronidazole	Ornidazole	Secnidazole	Tinidazole
**S(m)**	1.614	2.161	1.921	1.911
**S(a)**	1.634	2.159	1.887	1.923

**S value from Soczewiński's equation**	**Antihypertensive drugs:**			
	Nilvadipine	Felodipine	Isradipine	Lacidipine
**S(m)**	4.129	4.597	3.741	5.349
**S(a)**	5.330	4.841	4.715	5.690

**S value from Soczewiński's equation**	**Non-steroidal anti-inflammatory drugs (NSAIDs):**						
	Mefenamic acid	Indomethacin	Nabumetone	Phenylbutazone	Carprofen	Ketoprofen	Flurbiprofen
**S(m)**	2.879	2.773	3.456	2.682	2.854	2.139	2.568
**S(a)**	3.317	3.442	3.910	2.404	3.394	1.968	2.010

*where:

S(m) – S is the slope of the regression curie in accordance with Soczewiński-Wachtmeister's equation using methanol-water mobile phase

S(a) – S is the slope of the regression curie in accordance with Soczewiński-Wachtmeister's equation using acetone-water mobile phase

First group of drugs (antiparasitic drugs)

1. Metronidazole (2-Methyl-5-nitroimidazole-1-ethanol)

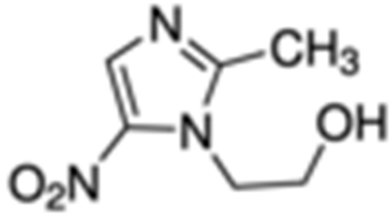

2. Ornidazole (1-(3-Chloro-2-hydroxypropyl)-2-methyl-5-nitroimidazole)

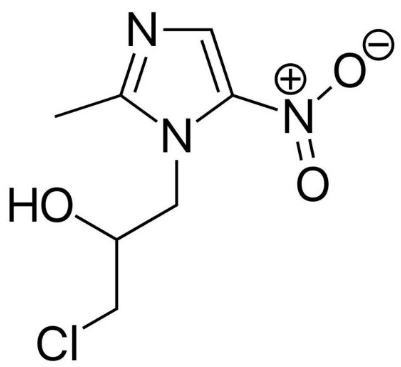

3. Secnidazole (1-(2-methyl-5-nitro-1H-imidazol-1-yl) propan-2ol, 1-(2Hydroxypropyl)-2-methyl-5-nitroimidazole)

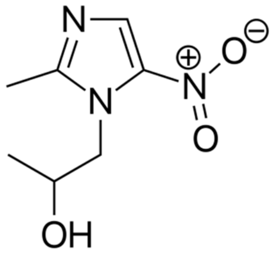

4. Tinidazole (1-[2-(Ethylsulfonyl)ethyl]-2-methyl-5-nitroimidazole)

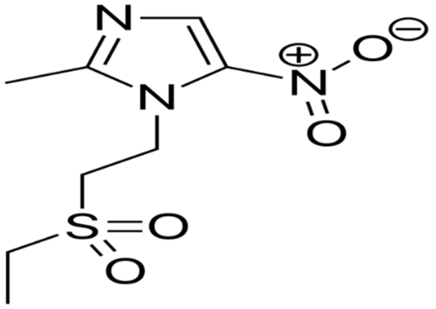

Second group of drugs (antihypertensive drugs)

1. Nilvadipine (2-Cyano-1,4-dihydro-6-methyl-4-(3-nitrophenyl)-3,5-pyridinedicarboxylic acid 3-methyl 5-(1-methylethyl) ester, 5-Isopropyl-3-methyl-2-cyano-1,4-dihydro-6-methyl-4-(m-nitrophenyl)-3,5-pyridinedicarboxylate, FK-235, FR-34235, Isopropyl 6-cyano-5-methoxycarbonyl-2-methyl-4-(3-nitrophenyl)-1,4-dihydropyridine-3-carboxylate)

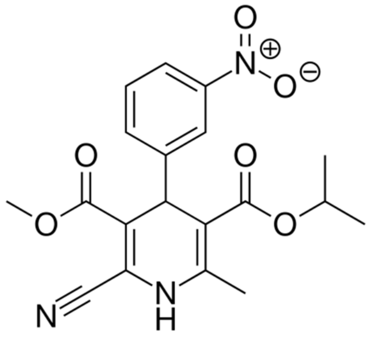

2. Felodipine (4-(2,3-Dichlorophenyl)-1,4-dihydro-2,6-dimethyl-3,5-pyridinecarboxylic acid ethyl methyl ester)

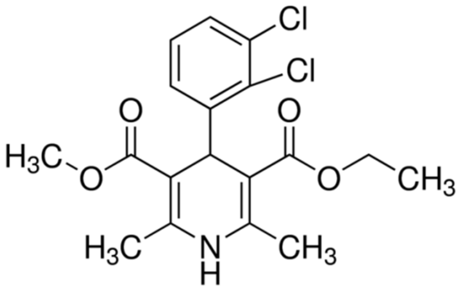

3. Isradipine (4-(4-Benzofurazanyl)-1,-4-dihydro-2,6-dimethyl-3,5-pyridinedicarboxylic acid methyl 1-methylethyl ester)

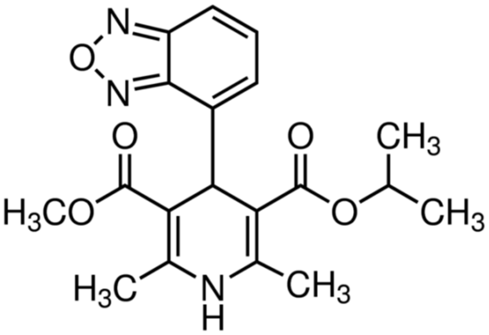

4. Lacidipine (3,5-Diethyl 4-{2-[(1E)-3-(tert-butoxy)-3-oxoprop-1-en-1-yl]phenyl}-2,6-dimethyl-1,4-dihydropyridine-3,5-dicarboxylate, 4-[2-[(1E)-3-(1,1-Dimethylethoxy)-3-oxo-1-propen-1-yl]phenyl]-1,4-dihydro-2,6-dimethyl-3,5-pyridinedicarboxylic acid 3,5-diethyl ester)

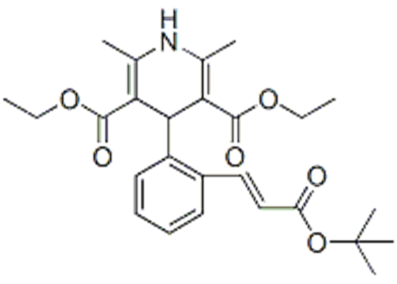

Third group of drugs (non-steroidal anti-inflammatory drugs)

1. Mefenamic acid (2-[(2,3-Dimethylphenyl)amino]benzoic acid, *N*-(2,3-Xylyl)anthranilic acid)

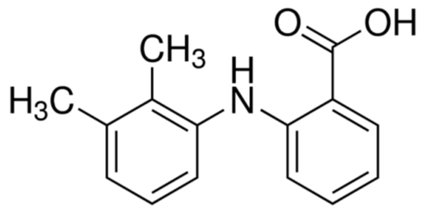

2. Indomethacin (1-(4-Chlorobenzoyl)-5-methoxy-2-methyl-3-indoleacetic acid)

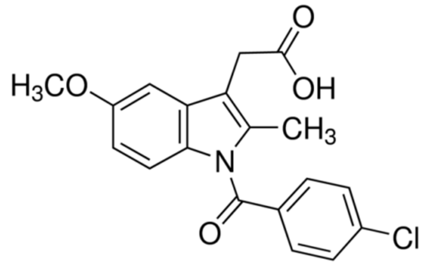

3. Nabumetone (4-(6-Methoxy-2-naphthyl)-2-butanone)

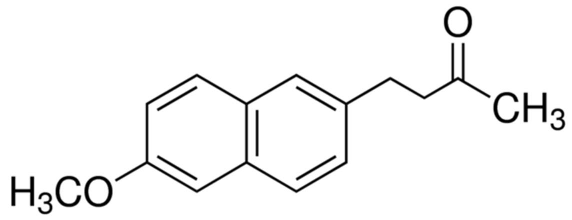

4. Phenylbutazone (4-Butyl-1,2-diphenyl-3,5-pyrazolidinedione)

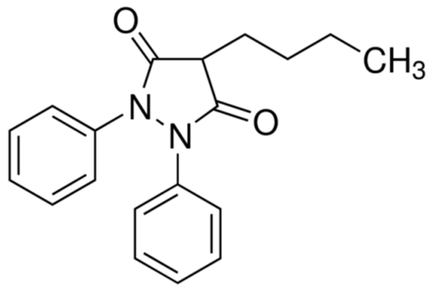

5. Carprofen (6-Chloro-α-methyl-9*H*-carbazole-2-acetic acid)

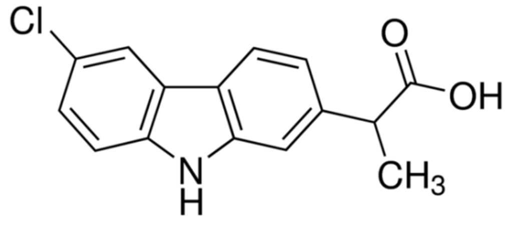

6. Ketoprofen (2-(3-Benzoylphenyl)propionic acid)

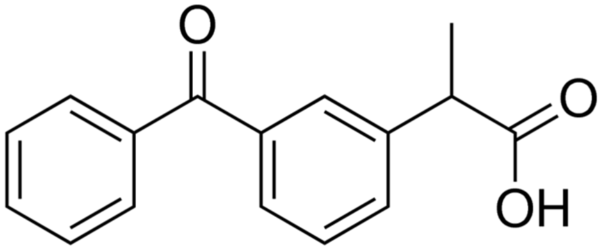

7. Flurbiprofen ((±)-2-Fluoro-α-methyl-4-biphenylacetic acid)

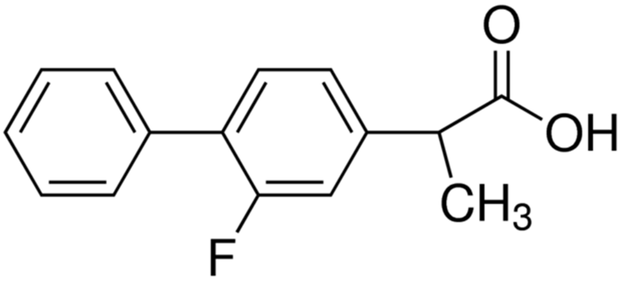

Supplement: Supplementary file 1 [file molecules-24-03187-s001.pdf]
